# Pharmacopœia Castrensis Ruthenica

**Published:** 1842-01

**Authors:** 


### Art. III.

1. *Pharmacopœia Castrensis Ruthenica.* Auctore Jacobo Wylie, Equite Baronetto, s.i.m. Archiatro, Doct. Med. et Chir. &c. Edit. Quart. Jussu Augusti Imperatoris.—*Petropoli*, 1840. 8vo, pp. 820.
2. *Pharmacopoea Danica, regia auctoritate a Collegio sanitatis regio Hafnensi edita.*—*Hafniæ*, 1840. 8vo, pp. 316.
3. *The Pharmacopœia of the Royal College of Physicians of Edinburgh.*—*Edinburgh*, 1839. 12mo, pp. 217.
4. *A Translation of the Pharmacopœia of the Royal College of Physicians of London*, 1836. *With Notes and Illustrations.* By Richard Phillips, f.r.s.l. & e., &c. *Fourth Edition, with additions.*—*London*, 1841. 8vo, pp. 456.
5. *New Remedies: the method of preparing and administering them; their effects on the healthy and diseased economy, &c.* By Robley Dunglison, m.d., sec. a.p.s., &c. *Third Edition, with numerous modifications and additions.*—*Philadelphia*, 1841. 8vo, pp. 541.
6. *The Elements of Materia Medica; comprehending the Natural History, Preparation, Properties, Composition, Effects, and Uses of Medicines. In Two Parts.* By Jonathan Pereira, f.r.s. & l.s. &c.—*London*, 1839-40. Two vols. 8vo, pp. 1500.

The composition of a pharmacopœia is obviously a matter of great national importance, inasmuch as it is the standard by which apothecaries and druggists are regulated in compounding medicines for the cure of diseases. The proper execution of such a work requires abilities of the very highest order; and our royal colleges, to their credit, have generally entrusted this to individuals distinguished for their scientific acquaintance with medicine. It is sometimes a difficult task, however, even for those who are gifted with the clearest insight into the medical profession, to determine whether a particular drug or formula ought to be admitted or excluded from the list, more particularly those that have been recently introduced into practice; for often it will be found that the virtues of new medicines are exaggerated by those who have been connected with their discovery, as a fond parent sees beauties in his own child which none other can discover. We therefore think that a prudent caution should be exercised by our medical authorities in not admitting new agents into the pharmacopœias until they have for some time received the stamp of respectable authority. This has in general been the rule; at the same time it must be acknowledged that undue attachment to some old friends has occasionally exceeded the bounds of an enlightened discretion.

In the construction of a pharmacopœia, a question will very naturally suggest itself to those engaged in the undertaking: What proportion of the agents or formulæ are employed by experienced and enlightened practitioners, by men who are capable of distinguishing between the *post hoc* and the *propter hoc*? A small proportion would be amply sufficient for the general necessities of patients, and there can be no doubt that the really well-informed part of the profession use comparatively few drugs and rarely complicated formulæ. At the same time it is obvious that if a pharmacopœia were contracted to such narrow limits, many substances would be excluded which have similar or analogous properties to those generally employed, and many formulæ for the same medicine would be omitted, which may be necessary for the more ready combination with others, or to suit the idiosyncrasies, or palate, or predilection of patients. It is therefore, upon the whole, better to have a variety of medicines which have the same or an analogous operation upon the system; but substances that are therapeutically inert, or that are even doubtfully so, should not be admitted. An investigation into the history of materia medica furnishes a lamentable example of the fallacious conclusions of professional men. Where are many of our celebrated specifics, our remedies for scrofula, cancer, phthisis, &c. which once figured so prominently in our medical works? Do not these diseases defy our art with as much pertinacity as they ever did?

If we examine the subject, it will be found that almost all the medicinal agents which have acquired a false celebrity, and which are now consigned to oblivion, have been indebted for their reputation to fortuitous circumstances; and it would be easy to show from the former history of diseases that the medicine employed in the treatment had no influence whatever in effecting the cure, but that the result was entirely owing to the efforts of nature. A physician, for example, employs a certain medicine in a few cases, and finds his patients recover; hence he concludes that the treatment and the cure stand to one another in the relation of cause and effect. Such a conclusion might be just in reference to actions taking place in inanimate matter, as in the domains of mechanics or chemistry; and hence chemical or mathematical physicians, although highly gifted in their peculiar sciences, are as apt to be misled in their medical and therapeutic conclusions as men of ordinary education. The analogy between the laws of dead and living bodies is often very remote: in the latter, vital processes are constantly producing changes, independent of the operation of any external agent; whereas in the former, with some exceptions, these changes are dependent upon the operation of external agents. Thus, if a certain agent is added to the urine after it has been evacuated for some time, it is fair to infer that the change produced is caused by its addition; whereas, if a certain medicine be prescribed to a patient, and the urine is found altered in its quality after its exhibition, it does not necessarily follow that these two circumstances are related to one another as cause and effect; and such a conclusion cannot be fairly drawn, until a uniform series of results have been obtained by a repetition of the experiment. Misapprehensions of this sort are the grand source of the introduction of all kinds of panaceas, and they are very frequently perpetuated by the flaming and exaggerated accounts of some writers and lecturers respecting the curability of all diseases by certain treatment, which produce a very false and injurious impression upon the minds of students, leading them, in their future practice, to bleed, blister, and treat heroically all affections that appear to be violent or intractable. There can be little doubt of the fact, that there are many diseases, among others our wide-spreading epidemics, for which there is no specific treatment, although in all, particular remedies, from the results of a limited number of cases, have been much vaunted. In such instances hygienic and palliative measures are much more successful, and their application requires more skill and discrimination in the medical attendant than the administration of particular medicinal agents or formulæ. These principles are too rarely acted up to by practitioners of medicine, who, while they admit their theoretical truth and accuracy, often neglect them in their practice, and they seem to have less engaged the attention of our framers of pharmacopœias than they ought to have done. In those works generally, and especially in these now before us, notwithstanding the great improvements in modern medicine, there are still many substances retained which are either altogether inert, or totally without therapeutic virtues, although in other respects active or even poisonous agents.

Before proceeding to an examination of the different works on our list, there are two preliminary points which ought to be adverted to, as various opinions are held upon these subjects in different countries. The Edinburgh college have followed the example of some of the continental and other colleges, in publishing their pharmacopœia in the vernacular tongue, while many other authorities still employ the Latin language.

It must be quite obvious that the vernacular language of any country will be better understood by the large majority of its practitioners and pharmacopolists than any dead or foreign tongue; and consequently that more accuracy will be secured in the preparation of the various formulæ, officinal or extemporaneous. This is an advantage, which in regard to the English language is very great, owing to the extent of its diffusion beyond Britain and Ireland, to our numerous colonies in the East, West, and South, and also to America. On the other hand it may be urged, that the Latin is or ought to be known by every well-educated practitioner in all countries, and that in this way it becomes an universal language, in which the pharmacopœias of any nation may be understood in all others. At one period this argument was employed to defend the publication in Latin, of all books on medical science; but in modern times, this practice has been almost totally abandoned, as a very impracticable, or at least a very inaccurate method of communicating knowledge. Translations have been found a much more convenient method of transferring the knowledge of one country to another.

It is unquestionably of great importance that medical practitioners should have a very liberal, classical, and scientific education; and the standard for this can scarcely be raised too high, if it be compatible with the means and circumstances of the candidate; but certainly there are no grounds for believing that the publication of a pharmacopœia in Latin would induce a student to learn this language, while there are so many translations to be had, unless there were some other reasons in operation. It is the duty of our teachers to make knowledge as easily procurable as possible, while it belongs to our examining boards to ascertain the classical, scientific, and medical acquirements of those who aspire to degrees in medicine.

We also agree with the Edinburgh college (and of course differ with Sir James Wylie and the London college), in their retention of the old names of many medicinal agents, as being less calculated to mislead those who are not very deeply versed in modern chemistry, and more particularly because our chemical nomenclature has of late been changed and modified so frequently. Besides, mistakes have sometimes occurred in consequence of the slight difference of name or their apparent identity, such as the bichloride of mercury for the chloride, the biniodide of mercury for the iodide, blunders which, it is unnecessary to show, might be as readily committed as they might prove highly dangerous. It is also evident, that though the ancient conventional names were authorized as the language which physicians ought to employ in their prescriptions, yet that this would not in the slightest degree prevent any one from becoming acquainted with the modern chemical or botanical name of any substance or preparation. But while we approve of the retention of the old names, at least until the chemical and botanical nomenclature shall be fixed upon a more permanent basis, we think it an affectation of simplicity to extinguish a scientific name because it is scientific, and substitute in its place one which is very indefinite or vulgar in its meaning. If a name be simple, not unwieldy in length, and be generally understood, it ought not to be changed into another which has a theoretical resemblance to other names in common use, for the very plain reason that it answers all the purposes intended, and its retention obviates all risk of mistake on the part of the practitioner or pharmacopolist.

We think the changes in the Edinburgh Pharmacopœia for the most part not objectionable, especially those where the old names are substituted for those previously last employed, but there are some exceptions. For example, *carbonas ferri precipitatus* is changed into *ferri ox. rubrum*, which might be mistaken for the red oxide of iron prepared by roasting the sulphate of this metal: the sesquioxide would have been a better name, as in the London Pharmacopœia. The term *cornu* is too indefinite, as it might apply to the horn of any animal, whereas it is the horn of the stag that is meant. *Cinchona coronæ* is not a happy change for the *flava* and *rubra* of the other kinds. Why is the adjective (cornararia) of this name not employed? Neither do we admire the alterations adopted in the case of the conserves of the gallic and canine rose, as they appear to be likely to lead to misinterpretation. The old names were much more definite, namely, *conserva rosæ caninæ*, and *conserva rosæ gallicæ*, than their corresponding new names, namely, *cons. rosæ fructus*, and *cons. rosæ*. Why was the old term *cons. cynosbati* not employed for the first, if the botanical name were considered objectionable? *Fæx sacchari* is far from being elegant, and such a name tends to support the opinion that molasses is merely dregs or scum of sugar, which is by no means true. This name appears to have been borrowed from the London Pharmacopœia. If the term *melassus*, adopted by Sir James Wylie, could have pleased the fastidious taste of the two colleges, it would have answered the purpose perfectly as an arbitrary word, and would have conveyed no false meaning like the one that has been adopted.

After these preliminary remarks, we proceed to make a few observations on some of the more leading or prominent substances, and on the manner in which they have been treated of or described in the various works under our present notice.

Hydrocyanic Acid. This acid has attracted great attention of late years, as well on account of its medicinal properties, valuable in some diseases, as from its rapid and fatal operation as a poison. When first introduced into practice, it was believed to possess an extraordinary amount of therapeutic virtue, being considered sedative, anthelmintic, carminative, diuretic, tonic, antispasmodic, &c.; but experience has shown that several of its vaunted virtues have not been genuine. It was at one period employed very extensively in pulmonary diseases, from the extravagant recommendations made of it by Majendie, Granville, &c., being by many supposed to be almost a specific in phthisis, asthma, hooping-cough, &c., but it is at present rarely prescribed in these affections, except as an elegant placebo to which past repute still attaches some dignity and importance. In affections of the stomach, however, it is sometimes a valuable remedy, particularly in that form of dyspepsia complicated with pain in the epigastrium. The profession are chiefly indebted to Dr. Elliotson for its administration in this form of disease, and for the first clinical illustrations of its efficacy. In vomiting arising from irritability of stomach or other causes, it very frequently succeeds in allaying the inverted action, although many practitioners have been more successful by the employment of creosote. Its external employment has been recommended in neuralgic affections, and in some impetiginous and papular eruptions, but its utility is very questionable if used in a very diluted state; and when applied in considerable doses to an abraded surface, it may be productive of great danger.

A very accurate and sound account of the therapeutic properties of this remedy is contained in Mr. Pereira's Elements. Dr. Dunglison enters very fully into the preparation, toxicological and therapeutic effects of this agent, but "he has not had sufficient reason to place it high in the rank of medicinal agents."

*Processes for obtaining Hydrocyanic Acid.* There is perhaps no medicinal preparation for which a correct and uniform process is so important as hydrocyanic acid. Fatal accidents have frequently occurred from its great variation in strength. It is not long since we ourselves had an opportunity of ascertaining that a nearly fatal result had occurred by the use of an acid four times stronger than the usual medicinal preparation, which was procured from a highly respectable druggist. The process generally employed for its production, and which is adopted in the London, Edinburgh, Russian, and Danish Pharmacopœias, is the decomposition of ferrocyanide of potassium by sulphuric acids. This is perhaps the best method of preparing it, although, if the operation is not carefully performed, a considerable portion of the acid, owing to its volatility, may escape condensation, and give rise to an uncertainty in its strength. The proportions of the salt and acid are however not the same in the different formulæ; hence the strength of the products must also be various. The proportions in the Edinburgh Pharmacopœia are, ferrocyanide of potassium 3 oz., sulphuric acid 6 fl. oz., water 16 oz., and the resulting product of hydrocyanic acid is 16 fl. oz. Those in the London Pharmacopœia are, ferrocyanide of potassium 2 oz., sulphuric acid 1½ oz., distilled water 1½ pint, and the product is 20 oz. In the Russian Pharmacopœia the quantities are, cyanide of iron and potass 2 oz., sulphuric acid 1 oz.; and the product is 20 oz. of acid, which is stated to contain 2 per cent. of pure acid. This last process is nearly the same as that in the London Pharmacopœia, the difference consisting in the proportions of sulphuric acid, and there certainly appears to be too little acid in the former.

The Danish process is of a similar kind, but alcohol is employed, which renders it more expensive than is necessary; for we believe that hydrocyanic acid, when prepared by sulphuric acid and ferrocyanide of potassium, keeps pretty well without any such addition. This is generally accounted for by the fact, that some of the sulphuric acid is distilled along with the hydrocyanic acid, and tends to prevent its decomposition. Mr. Phillips states that the addition of a little muriatic acid retards its decomposition.

From the variety of processes, it must be obvious that the strength of the different products cannot be uniform; and though this discrepancy might be obviated to a certain extent by a table of doses, it is to be regretted that the plan of giving these is not adopted in the British Pharmacopœias, although the omission of the London college is well supplied in Mr. Phillips's translation. In the Danish Pharmacopœia there is a posological table of the more potent remedies, and in the Russian Pharmacopœia the doses are given in the general description of the agents.

There is another process for preparing hydrocyanic acid, first proposed, we believe, by Dr. Clarke, and afterwards modified by Mr. Laming, which is occasionally employed by pharmacians, namely, the decomposition of cyanide of potassium by tartaric acid. When a pure cyanide of potassium is used, a very excellent product for practical purposes is the result of this simple method. It contains no doubt a minute portion of bitartrate of potass, but this is not at all injurious for medicinal use. Mr. Laming's addition of a little alcohol is an improvement, in as far as it tends to prevent decomposition.

*Cyanide of Potassium.* This salt, if it could be readily obtained pure, would, in our opinion, supersede in a great measure the employment of hydrocyanic acid, for its action on the system is exactly the same, and its use would obviate the accidents which too frequently result from the uncertainty in the strength of the former. It is liable, however, to deliquesce when not carefully excluded from the air, and then loses its cyanogen, which of course will render it more or less inert; but there is not much greater difficulty in preserving it than in preventing the volatilization of hydrocyanic acid. Its employment would also be attended with this important advantage, that no mistake could ever occur in prescribing it; for a given weight could not contain more than its atomic proportion of cyanogen, although if spoiled it might contain too little. It is surprising that neither the London nor the Edinburgh Pharmacopœia contains a formula for its preparation, although the former contains one for the bromide of potassium, an agent whose medicinal properties have by no means been ascertained. The Danish College seem also to consider it unworthy of insertion; but the author of the Imperial Pharmacopœia, being more alive to the modern discoveries in medicine, has given it a place, although it is not recommended very strongly. The method employed for its preparation is to calcine the ferrocyanide of potassium in an earthen retort, and after the process is finished, to remove the upper stratum of the saline matter, which is of a white colour and "concrete like glass." We question if this can be pure cyanide of potassium.

*Laurel Water.* This preparation is generally employed on the continent; and there is a formula for its preparation in the pharmacopœias of both Russia and Denmark, which is nearly the same. It has also been introduced into the Edinburgh Pharmacopœia. We believe it is not liable to decomposition, but it must necessarily be uncertain in the proportions of hydrocyanic acid which it contains. The dose is stated in the Russian Pharmacopœia to be from 10 to 30 drops, while in that of Denmark the maximum is as high as 60 drops.

*Aconitine* (*aconitina*). A formula for the preparation of this proximate principle has been admitted into the London Pharmacopœia; but neither the Edinburgh, nor Russian, nor Danish colleges have sanctioned its introduction, a caution which we approve of. It has no doubt been ascertained to be extremely poisonous to animals; but on this account more circumspection ought to be exercised in recommending it, until its therapeutic properties have been well ascertained. We are indebted to Dr. Turnbull for its introduction into practice, and the proofs of its utility have been chiefly collected by him; but we require to have his alleged results confirmed by a more extended clinical experience. In the Russian Pharmacopœia it is shortly described under the name *aconitia,* and is said to have "the form of acicular yellow transparent crystals, which have a very bitter taste, and are soluble in cold water and boiling alcohol." (p. 9.) The following is a part of Mr. Pereira's excellent and full account of this agent:

"As prepared by Mr. Morson, this substance presents the following properties: It is a white, odourless solid, either dull and amorphous or somewhat sparkling, and apparently crystalline. .......... Heated in a tube, aconita readily fuses, and forms a pale amber-coloured liquid; and at a higher temperature decomposes. It is not volatile. Heated on platinum, or over a spirit lamp, it is speedily and entirely dissipated. It is soluble in alcohol, ether, and the acids. From its acid solution it is precipitated by ammonia........... Mr. Morson's aconitina is so powerful that one fiftieth of a grain has endangered the life of an individual. It is the most virulent poison known, not excepting hydrocyanic acid. .......... A minute portion of an ointment, composed of a grain of the alkaloid to two drachms of lard, applied to the eye, causes almost insupportable heat and tingling, and contraction of the pupil. This last effect was shown me by Dr. Turnbull, in some amaurotic cases of several years' standing, and whose pupils underwent no change when the eye was exposed to strong daylight. In very minute doses it has caused heat and tingling upon the surface of the body, and sometimes diuresis; but it cannot be administered internally with safety...........Of the great efficacy of aconitina in neuralgic and rheumatic affections no one can entertain any doubt who has submitted the remedy to trial. The following are Dr. Turnbull's formulæ for using aconitina externally:

"1. *Unguentum Aconitinæ. Aconitine Ointment.* (Aconitina, gr. xvj.; Olive Oil, ʒss.; Lard, ℥j. Mix.) It is employed by friction, with the finger, during several minutes.

"2. *Solutio Aconitinæ. Aconitine Embrocation.* (Aconitine, gr. viij.; Rectified Spirit, ℥ij. Dissolve.) Used by friction-sponge (as a sponge tooth-brush). Care must be taken not to employ it where the skin is abraded." (pp. 1338, 1343-4.)

*Tartarized Antimony.* (Antimonii Potassio-Tartras. *Lond.* Antimonium Tartarisatum. *Ed.* Tartras Stibii et Potassæ. *Russ.* Tartarus Stibiatus. *Dan.*) This salt is universally acknowledged to be a most valuable medicine, and is perhaps more frequently used in febrile and inflammatory diseases than any other in the materia medica. The Edinburgh College have changed their process for making it. Formerly they employed the *crocus,* but now use the sesquioxide of antimony, prepared by decomposing the chloride of this metal by mixing it with a large portion of water. It is similar to the process of the Dublin Pharmacopœia, and is certainly much better calculated to furnish white crystals than the employment of the crocus is. The method adopted in the Russian Pharmacopœia is very similar to that of the Edinburgh College; but in the Danish the gray oxide of antimony is employed

*Tests of Purity.* According to the Edinburgh College, tartarized antimony is "entirely soluble in twenty parts of water: solution colourless, and not affected by solution of ferrocyanide of potassium: a solution in forty parts of water is not affected by its own volume of a solution of eight parts of acetate of lead in thirty-two parts of water and fifteen parts of acetic acid." Fifteen parts of water are generally reckoned sufficient to dissolve this salt, as stated by Mr. Phillips. The acetate of lead was originally suggested as a test by M. Henry, who states, in addition, that it is not precipitated by muriate of baryta, neutral oxalate of ammonia, and acidulous nitrate of silver. In the Russian Pharmacopœia it is merely stated to be soluble in fifteen parts of cold and in two of boiling water, that it is decomposed by mineral acids but not by the acetic, and that a polished plate of iron attracts the antimony. The Danish College again consider it impure if not completely soluble in fifteen parts of water, and that no blue colour or precipitate should be produced by the ferrocyanide of potash, which is intended, we presume, to indicate the presence of iron; and that no green colour should result from the addition of the liquor cupri ammoniacalis, which if produced might indicate arsenic.

*Nitrate of Silver.* This salt is one of the most useful of external applications, particularly in the healing of ulcers, and also in subduing cutaneous inflammation. Mr. Higginbottom first drew the attention of practitioners to its use in arresting phlegmonous inflammation, and in checking the progress of erysipelas; and subsequent experience has proved satisfactorily to many, that its powers are not less remarkable in these diseases than they are in controlling the inflammatory affections of mucous membranes. The employment of the nitrate of silver in these cases, as well as in diseases of the eye, ulcers, cutaneous diseases, &c. is well known; but there are some other affections in which we have found it extremely valuable, although, we are persuaded, this practice is not generally known or acted upon.

*a.* The analogy between *Gonorrhœa* and purulent ophthalmia might very naturally lead a practitioner to employ nitrate of silver in the cure of the former, if he reasoned at all correctly in the matter; for they are both purulent inflammations of mucous membranes. Many of the agents used as injections in gonorrhœa, such as the sulphate of zinc and copper, acetate of lead, bichloride of mercury, sulphate of alumina, &c. excite more or less inflammatory action in the urethra, and in scrofulous subjects sometimes do more harm than good; but the nitrate of silver, in place of exciting more, generally diminishes inflammation, and on this account may be employed at the very commencement of the disease. We have used this remedy for the last ten or twelve years with much more success than any other injection, and have had no reason to believe that it causes hernia humoralis or any other bad consequence. The proportions we employ are from 1 to 4 grains to an ounce of water, the strength being regulated according to the greater or less inflammation of the parts.

*b.* The tincture of camphor is so generally reckoned a sovereign cure for *Chilblains*, that many practitioners would think it heretical to use any other remedy. As far as our experience goes, this agent very often fails in affording relief, and is less effective than the nitrate of silver. A pretty strong solution of this is required, the strength being varied from 10 to 30 grains of the lunar caustic to an ounce of water. Occasionally we have used the proportion of a drachm to the ounce of water, and even more. In one obstinate case, we used a saturated solution (equal parts of nitrate of silver and water), which produced a permanent cure.

*c.* An ointment made from galls is a favorite remedy for *Hemorrhoids*, but, like camphorated spirit of wine in the cure of chilblains, it often fails. An ointment composed of from 5 to 10 grains of nitrate of silver, very finely pulverized, and an ounce of lard, succeeds in many cases where the hemorrhoids are recent. When hemorrhage arises from internal piles, or from congestion of the lining membrane of the anus, the solution, in the proportion of from 10 to 30 grains to an ounce of water, injected with a syringe, is a preferable method. It ought, however, to be remembered that when the solution is strong, not more than a drachm of it should be thrown into the rectum. In prolapsus ani the efficacy of this solution is not so certain, although we have on several occasions succeeded in curing the patient with it.

The external employment of nitrate of silver is shortly but generally described in the Russian Pharmacopœia, and its employment in checking erysipelas by the formation of a ring with caustic is also noticed as the result of recent experiments. Its employment in chronic blenorrhœa, leucorrhœa, and in excoriated nipples is also mentioned.

*Chloride of Lime.* This agent, as is well known, is now extensively employed in the bleaching of cotton and other cloth, and its application for this purpose has been of immense value to the manufacturer in expediting his processes. It has of late years also been introduced into medical practice, and has been employed in various diseases as well as for the purpose of destroying contagious and fetid effluvia. As an application to ulcers, a solution of this substance is often of considerable service by inducing, like many other caustic substances, a more healthy action in the parts. It is also stated by several authorities, that it is successful in the cure of scabies and other skin diseases, either in the form of ointment or solution. It has been recommended in ophthalmia, and Mr. Pereira states that he has found a weak solution of the chloride very successful in the purulent ophthalmia of infants. We would prefer the solution of nitrate of silver, as it rarely fails. It forms a very valuable gargle in fetid ulcerations of the gums, mouth, or fauces, whether arising from common causes or from the use of mercury, in the proportion of four to six grains of the chloride to an ounce of water. It has been employed internally in some of the contagious fevers, such as scarlatina and typhus, and experimenters report favorably of its effects in improving the secretions and in destroying their fetor. It ought, we think, to be employed in very small doses, if employed at all in these affections, for it is very irritating in its effects on mucous surfaces. We agree with Mr. Pereira in his opinion respecting the inefficiency of the chlorides to destroy contagious effluvia.

"The power of the chlorides (hypochlorites) to destroy infection or contagion, and to prevent the propagation of epidemic diseases, is less obviously and satisfactorily ascertained than their capability of destroying odour. Various statements have been made by Labarraque and others (vide Alcock's Essay, p. 55, et seq.) in order to prove the disinfecting power of the chlorides with respect to typhus and other infectious fevers. But, without denying the utility of these agents in destroying bad smells in the sick chamber, and in promoting the recovery of the patient by their influence over the general system, I may observe that I have met with no facts which are satisfactory to my mind as to the chemical powers of the chlorides to destroy the infectious matter of fever. Nor am I convinced by the experiments made by Pariset and his colleagues (*Bullet. des Sciences Méd.* xix, 233) that these medicines are preservative against the plague." (p. 352.)

In the Russian and Danish Pharmacopœias, a formula is given for the chloride which is named calcaria chlorata, chloretum calcis, &c., while in the Edinburgh, it is merely inserted in their list of materia medica; and the London formula is so indefinite that no one would undertake to form it thence. In this country there is no occasion for any formula in our Pharmacopœias, for manufacturers of this article are in general well acquainted with the mode of making it of good quality; but it appears that in Russia it contains too much lime. In the Russian Pharmacopœia a pretty good account is given of its therapeutic properties, and it is also recommended in the form of lotion for fetid perspirations of the feet, pruritus of the vulva, the destruction of lice and fleas, and also to tobacco-smokers for removing the nicotian fetor of their breath, "necnon iis, qui, fumo nicotianæ imbuti spiritum edunt ingratum." This is a valuable hint to the nicotian criminals for preventing discovery.

*Gamboge.* Gamboge is a purgative which is not often employed uncombined in medical practice, on account of its irritating, drastic, and dangerous qualities, in justification of which reserve we have lately had some lamentable instances of its ill consequences in the case of *Morison's pills.* It may, however, be used quite safely as a component part of purgative pills, if very finely pulverized and in minute proportions, as in the Pil. Cambog. comp. of the London Pharmacopœia. Some apothecaries occasionally use it as a cheap substitute for a portion of the scammony in the composition of the compound colocynth pill of the Edinburgh College. In the Edinburgh Pharmacopœia two kinds of gamboge are noticed, viz. the Siam and the Ceylon, which are nearly identical in chemical composition, although the first is almost the only one known to druggists. The chemical characteristics of this agent are very little known to medical men, as it is only of late years that they have been much investigated; although it is highly important that they be generally understood, for this dangerous purgative is not only employed extensively by empirics, but is also used to colour confectionaries. The following are its chemical characteristics as given by Mr. Pereira:

"Gamboge emulsion becomes transparent and deep red on the addition of potash, forming gambogiate of potash. Digested in alcohol or ether, gamboge yields orange-red tinctures, (solutions of gambogic acid.) The etherial tincture dropped on water yields, on the evaporation of the ether, a thin, bright yellow opaque film or scum (gambogic acid), soluble in caustic potash. The alcoholic tincture dropped into water yields a bright, opaque, yellow emulsion, which becomes clear, deep red, and transparent on the addition of caustic potash. The gambogiate of potash (obtained by any of the above processes) gives, if the alkali be not in excess, with acids, a yellow precipitate (gambogic acid); with several metallic salts, as acetate of lead, nitrate of silver, &c., yellow precipitates (metallic gambogiates); with sulphate of copper, brown (gambogiate of copper); and with the salts of iron, dark brown (gambogiate of iron)." (p. 1230.)

The test of the Edinburgh College is the tincture of iodine, but it is only capable of detecting its adulteration with starch, which is occasionally contained in common gamboge.

*Cantharides.* In the Russian Pharmacopœia it is stated that the "lytta vesicatoria abounds in the southern provinces of Russia, but generally inhabits Egypt and the warmer regions of Europe." According to Robiquet cantharides are composed of cantharidin, a green fixed oil, fatty matter, ozmazome, a black matter soluble in water, a yellow matter soluble in ether and alcohol, acetic and uric acids, phosphates of lime and magnesia. The cantharidin is the vesicant principle, crystallizing in micaceous plates, which are fusible, forming a yellow oil. It is extremely active even in minute quantities, and the 1-100th part of a grain is capable of producing vesication upon a tender surface, such as the lip. The expense of this proximate principle has operated as an objection to its general employment as a vesicatory; but Messrs. Smith of Edinburgh have succeeded in obviating to a great extent this drawback. It is stated that their *tela vesicatoria* is formed by spreading an ethereal extract of cantharides upon cambric paper, and we believe it is now very frequently employed in place of the empl. cantharid. It possesses the advantages of being light, elegant, and even attractive in its appearance, and, as far as our experience goes, generally produces complete vesication. We cannot, however, agree with the statement that it never causes strangury. Owing to the thin quality of the paper, it is in our opinion more difficult to keep in proper apposition than the ordinary plaster.

In some diseases a more rapid vesication is required than is produced by blistering plasters. The acet. cantharid. seems to be well adapted for this purpose; it has accordingly been introduced into both the London and Edinburgh Pharmacopœias. The addition of the euphorbium in the latter is certainly no great advantage, for it possesses no superiority over cantharides as a vesicant, if it be even equal to it; and if the proportion of the latter was too small, why not increase it? As it is, however, this preparation ought to be superior in efficacy to that in the London Pharmacopœia; it is not contained in either the Russian or Danish publications.

There are two points connected with the employment of blisters, which it may be useful here to refer to, viz. the methods recommended for preventing strangury, and the plan which ought to be pursued in those habits in which a gangrenous ulceration is likely to occur. Various substances, such as opium, balsam of Peru, camphor, sulphuric or nitric acid, have been used as an addition to blistering plaster, in order to prevent strangury, but we believe with very little effect. In the Russian Pharmacopœia, a piece of muslin moistened with vinegar (nebula lintea) is recommended to be placed upon the plaster, a practice which is occasionally employed in this country, and the utility of which is by no means imaginary, although it sometimes prevents thorough vesication. The practice of dusting powdered flies upon the plaster immediately before its application is injurious, in so far as the loose particles are apt to get entangled with the skin, and are thereby more liable to be absorbed. We have observed that when the cantharides are coarsely pulverized or imperfectly combined with the fatty bodies, that strangury is more apt to occur; and in proof of which we may quote the fact known to many practitioners, that the unguentum infusi cantharidis of the Edinburgh Pharmacopœia is less liable to occasion painful micturition than the unguentum cantharidis of the same. This, we think, may be accounted for on the principle that the extract of the flies in the one case is minutely and intimately combined with the cerate; while in the other it is less so, and is thereby more readily absorbed.

The *unguentum cantharidum* of the Danish Pharmacopœia appears to be a good formula; in this the cantharidin or vesicant principle is first dissolved in olive oil in place of water, as recommended in other similar works. We should think it little liable to excite strangury, on account of the minute combination of the cantharidin with the fatty matters.

Blisters are disapproved of in several diseases by authors and practitioners where there is a tendency to gangrene. In the Russian Pharmacopœia it is stated that "the use of blisters is injurious in all diseases where there is any fear of gangrene occurring; for example, in the last stage of petechial fevers, malignant synocha, malignant smallpox, and also in dropsy and erysipelas which has a gangrenous tendency." (p. 248.) There can be no doubt that gangrene does occur occasionally in some of these diseases, particularly in malignant measles; but it is equally true that vesicatories are often very advantageous in the treatment, particularly when internal inflammations occur; and on this account they ought not to be proscribed, if there be any method of avoiding the evil consequence.

The remarks of Mr. Pereira on this subject agree with our own experience:

"It is hardly safe to apply blisters to children immediately after exanthematous diseases, sloughing being not an unfrequent result. If it be required to produce in them counter-irritation, the best plan is to dilute the common blistering plaster, by mixing it with three times its weight of soap cerate. I have seen this compound frequently employed, but never observed any unpleasant results from it. Another plan, sometimes adopted, is to apply a common blister for an hour or two only, so that it shall merely produce rubefaction." (p. 1372.)

It is generally found that though rubefaction only be the result after the removal of the plaster at the end of two hours, yet that by the application of a poultice, more or less vesication makes its appearance in a few hours afterwards. There is another vesicant, however, which we have repeatedly used in malignant measles and scarlet fever, viz. the nitrate of silver, and we have not, in any of the instances where it was employed, seen gangrene induced in the resulting blister.

*Irish or Carrageen Moss*, (Chondrus crispus.) This fucus is found abundantly on the coasts of Ireland; and within the last ten or twelve years has attracted some attention as a substitute for arrow-root, sago, &c. It has, however, been long known in Ireland, and highly esteemed by the peasantry on the western coast as a nutritive article of diet and as a remedy for consumption, diarrhœa, scrofula, and diseases of the kidney. Mr. Pereira gives a short account of its properties and uses, a portion of which we shall lay before the reader, as no account of this vegetable is to be found in our ordinary works on materia medica.

"In the recent state it is purple-brown or purple-red, becoming greenish and untimely whitish in decay. As met with in commerce it is dry, crisp, mostly yellowish or dirty white, but intermixed with purplish-red portions, inodorous or nearly so, with a mucilaginous taste. It swells up in water. A calcareous meshy crust (consisting of various species of Flustra) is frequently found on the frond. ........ It is usually exhibited in the form of decoction or jelly.

"1. *Decoctum Chondri.* Macerate half an ounce of carrageen in cold water, during ten minutes; then boil in three pints of water, for a quarter of an hour. Strain through linen. Milk may be substituted for water when the decoction is required to be very nutritious. By doubling the quantity of carrageen a mucilage is procured. Sugar, lemon-juice, tincture of orange-peel, or aromatics, as cinnamon or nutmeg, may be employed as flavouring ingredients.

"2. *Gelatina Chondri.* Prepared by concentrating the decoction, or by employing a larger quantity of carrageen." (pp. 563-4.)

The following is M. Beral's formula for preparing what he is pleased to call analeptic or restorative milk, which perhaps may be interesting to some of our dietetic practitioners: Take milk 24 ounces, Irish moss 4 scruples, sugar $\frac{1}{2}$ ounce, canella or cinnamon 1 scruple, macerate the moss in cold water for a few minutes, then shake the water out of it, and boil until the liquid attains the consistence of warm jelly; when cold it has the consistence of cream. In the Danish Pharmacopœia the proportions given for making "Carraghen-Mos-Gelee" are 2 drachms of the moss to 12 ounces of milk. A very good account of this article is given by Dr. Dunglison, and the formulæ of several authors are appended.

*Iceland Moss,* (Cetraria islandica.) Mr. Pereira's description of this moss is short and good, but, both in his account of it and Irish moss, his dietetical remarks are rather imperfect. It has been estimated that a ton of Iceland moss contains about as much nutritive matter as half a ton of wheat, and Professor Burnet states, in his Medical Botany, that the Saxon government published a report upon this subject a few years ago; in which we are informed that 6 pounds and 11 ounces of lichen meal, boiled with fourteen times its quantity of water, and baked in this state with $39\frac{1}{2}$ pounds of flour, produced $111\frac{1}{2}$ pounds of good household bread. Without this addition, the flour would not have produced more than $78\frac{3}{4}$ pounds of bread, consequently the addition of 6 pounds and 11 ounces of lichen meal occasioned an increase of $32\frac{5}{6}$ of good bread. Bread manufactured in this manner would of course, like sago bread, contain a larger proportion of water in proportion to its weight than common wheaten bread. The method recommended by Mr. Pereira for extracting the latter principle is the one generally practised; but his proportion of the carbonate of potass is too small, viz. one part of this salt to 300 of water. If the carbonate of potass be rendered alkaline by lime, a much less proportion of it will be required; and we have found that four or five parts of this salt treated in this manner are sufficient to extract completely the bitter principle, if maceration for eight or ten days be employed. The lichenous odour of this substance, like that of the Irish moss, is much lessened by maceration in a weak solution of chloride of lime.

The proximate or bitter principle cetrarin has lately been employed in the cure of intermittents, and is described by Dr. Dunglison as one of the new remedies. This author states that it has the form of a white powder, which sometimes presents the appearance of small globules. Its dose is about two grains, and he calculates that its price must be less than that of quina, as M. Herberger obtained 135 grains from a pound of Iceland moss.

*Balsam of Copaiva.* The first notice of copaiva balsam, as well as of the tree yielding it, was given by Piso in 1648. Mr. Pereira enumerates four species from which it is obtained. It is principally obtained from Para and Maranham, but also from Carthagena, Maracaibo, and Savanilla, and occasionally from Rio Janeiro.

*Adulteration.* Mr. Pereira remarks that "there is no reason to suppose that balsam of copaiva is adulterated in this country now; though the fact mentioned by Dr. Paris (Pharmacologia, ii. 183, 6th ed.) proves that formerly it was: 'A curious trial took place some time since, between the owners of certain premises that were burnt down, and the Governors of the Sun Fire Office, in consequence of the latter refusing to indemnify the proprietor for his loss, because the fire had been occasioned by his making Balsam of Copaiba.'" (p. 1174.) We beg leave to differ in opinion from the highly respectable author on this point, for we have known balsam of copaiba adulterated, not only with fixed oils, such as rape oil, but also with Venice turpentine, although the adulteration is not so frequent as it once was. The Edinburgh College give the following as tests of its purity:

"Transparent: free of turpentine odour when heated: soluble in two parts of alcohol: it dissolves a fourth of its weight of carbonate of magnesia, with the aid of a gentle heat, and continues translucent." (p. 16.)

Its solubility in alcohol would detect the presence of almost all the ordinary fixed oils; but we are satisfied that a certain proportion of castor oil might be present, which being soluble in the alcohol, might escape detection by the carbonate of magnesia. The evaporation of a drop of the suspected balsam upon a piece of unsized paper, as recommended by Mr. Pereira and as occasionally practised, ought to have been added. If the balsam be pure a resinous spot is left; but if it be adulterated with a fixed oil, it is greasy and soft. The action of sulphuric acid upon it is also worthy of trial in doubtful cases. When three drops of pure balsam and one of sulphuric acid are well mixed together, they unite into a plastic reddish mass; while if it be mixed with castor oil to any considerable extent the mixture remains fluid. Venice or other species of turpentine are the most difficult of detection, and the mode recommended by the Edinburgh College is the only one that has hitherto been practised or recommended; but it is by no means to be depended on, for the odour of the balsam is so strong and it is volatilized so readily, that it completely conceals the odour of the former, unless it be present in large proportion.

Mr. Pereira, in describing its physiological operation, refers to the eruption occasioned by its internal employment, a circumstance which has attracted some attention of late years. He says that "not unfrequently it gives rise to an eruption, usually of a scarlet colour, referrible to either urticaria or erythema, though some describe it as being miliary. Vesicular eruptions are also spoken of, but I have never seen them. Mr. Judd has depicted two eruptions caused by the balsam: one he calls small puniceous patch eruption, the other was a papular eruption. Rheumatism has also been ascribed to the use of the balsam (Br. and For. Med. Rev. vol. viii. p. 280; and Lancet, vol. ii. for 1837-8, p. 826.") (p. 1176.) The precise character of this eruption has not hitherto been determined, and we have no doubt from the discrepancy which exists respecting it, that cutaneous affections which were owing to other causes have in many instances been confounded with it. This subject is, therefore, worthy of further investigation by some competent person.

*Administration.* There are two essential points connected with the exhibition of copaiva, viz. 1, the most efficacious formula; and 2, the one which is most easily taken, and which produces the least inconvenience to the patient afterwards, for we know no medicine which so universally excites disgust when a perseverance in its use is required. In our experience the most efficacious formula is an emulsion made with gum arabic water, and a few drops of aq. potassæ, to which may be added a little sugar and spirit of nitrous ether. Mr. Pereira gives very full instructions on this head, to which we refer our readers. The gelatine capsules of copaiva have obviated one of the great objections to its continuous employment, for they have neither the odour nor taste of this balsam. "These capsules were the invention of a Frenchman of the name of Mothe. They are now manufactured in London by Mr. Wildenow, whose agent informs me that the capsules are made of a compound of gelatine, gum, and sugar. In very hot weather some difficulty is experienced in making them. Desfontenelles has described a method of making the capsules. It consists in coating the distended and greased swimming bladder of the tench (or other small fish) with gelatine, and afterwards withdrawing the air and the membranous mould. Manufacturers, however, must have some cheaper and more readily procurable mould than a fish's swimming bladder. Gelatinous capsules of cubebs, containing the oil of cubebs, are also prepared by Mr. Wildenow." (pp. 1179-80.) The same author remarks that the resin is the least active part of the balsam, and he prefers the oil, in the dose of from ten to twenty drops, which may be gradually increased. The Edinburgh College have introduced a formula for its preparation into their Pharmacopœia, but give no test for its purity; and perhaps there is none known that can be depended on.

*Creosote.* We shall assume that the general qualities and administration of this medicine are pretty well known, and will therefore only allude to one or two points connected with its therapeutic agency. As an internal remedy we consider creosote chiefly valuable for its powers in checking vomiting, arising from irritability or derangement of the digestive organs, and we have tried it so extensively as completely to satisfy ourselves upon this point. It would be an exaggeration to say that it scarcely ever fails; but certainly it succeeds more frequently than any other agent we have tried in such cases. We agree with Mr. Pereira in thinking that it frequently fails where the vomiting depends upon organic disease, but even in some of these cases, a temporary relief is obtained from it. There are some essential oils which are also very efficacious in checking vomiting, such as the oil of cloves, which we have often employed, particularly in affections of the digestive apparatus occurring in children, who could not be induced to swallow creosote. The formula we employed is 8 drops oil of cloves, 2 drops liq. potass., 30 drops of sp. æth. nitros., 2 ounces syrup, rubbed up together in a mortar. Occasionally the addition of a little magnesia, laudanum, or tincture of hyoscyamus is useful, if there be acidity, diarrhœa, or pain from flatulency. Dr. Elliotson and others have recommended creosote in the treatment of diabetes. We have tried it both in the saccharine and insipid form of the disease, and, as far as our experience extends, with very little effect in altering either the quantity or quality of the urinary secretion. As an agent for checking hemorrhage it is worthy of a more extensive trial than it appears to have received from the profession. In such cases it appears to act principally by coagulating the albumen of the blood, and thus forming a solid barrier to its impetus, nearly on the same principle as nitrate of silver acts, by the formation of a slough.

Creosote (*Kreosotum*) does not seem to be much used in Denmark, as it is very briefly noticed in the Pharmacopœia of that country. A pretty good article on this agent is, however, to be found in that of Russia, accompanied with copious details of the process for its preparation. In this work it is recommended as an application to chilblains, either recent or ulcerated, in the form of liniment made with olive oil, in the proportion of 5 to 10 drops to an ounce of oil. A dilute solution is stated to be of service in menorrhagia, when injected into the vagina. In Dr. Dunglison's work a very complete and admirable account is given of the properties and therapeutic agency of this article.

*Croton Oil.* This oil is now well known as a powerful cathartic in cases of obstinate constipation, in apoplexy, in diseases accompanied with a comatose or delirious state, tetanus, mania, &c., its small dose, independent of other advantages, rendering it comparatively easy of administration to persons who are either unable or unwilling to swallow bulky medicines. In cases of constipation accompanied with vomiting, its combination with opium is often advantageous, exhibited in the form of a pill, viz. two or three drops of croton oil and one grain of opium. The opium tends to relieve the vomiting, but does not prevent the purgative operation of the oil, which generally takes place in a few hours after the dose is swallowed. We have found the oil a valuable addition to the compound colocynth pill as a laxative in very obstinate conditions of the bowels, in the proportion of one eighth of a drop to each pill. As an external agent, however, for producing a rubefacient or pustular effect, it has not come into general employment, as its price is high and its operation not superior to that of many other agents. In the Russian Pharmacopœia there is an excellent article on croton oil, and a curious and agreeable mode of exciting purgation is stated to be practised in Asia and Oriental India, viz. to macerate an orange or lemon in the oil for a month and then to apply it to the nostrils, "ut ex olfactu ventris exoneratio insequatur . . . . quod tamen (the author adds,) incredibile est auditu."

*Test of Purity.* In the Russian Pharmacopœia it is stated to be "often adulterated with ol. ricini, ol jatrophæ curcas, ol. jatrophæ multifidæ, or any other expressed oil; nay it is made factitiously from the resin of jalap dissolved in Canada balsam, which, however, may be detected by the aid of alcohol extracting the very acrid resin. In France the oil of euphorbia lathyrus is substituted for croton oil, or is used for its adulteration." (p. 136.)

In the Edinburgh Pharmacopœia the following tests are given :

"When agitated with its own volume of pure alcohol and gently heated, it separates on standing, without having undergone any apparent diminution." (p. 17.)

It seems implied in the above directions, if we understand them correctly, that the oil is not permanently soluble in pure alcohol, for it separates by the aid of heat, without any diminution. Dr. Nimmo many years ago proposed its partial solubility in alcohol as a test of its purity; and Mr. Pereira states that it is soluble in this fluid, the same fact being also mentioned by Berzelius. Dr. Dunglison mentions that it is almost wholly soluble in absolute alcohol.

*Cubebs.* Mr. Pereira is of opinion that this pepper was not known to the ancient Greeks, although this has been repeatedly stated by authors. He mentions, however, its use in England 500 years ago, "for in 1305 Edward I. granted to the corporation of London the power of levying a toll of one farthing a pound on this article in its passage over London Bridge."

Mr. Pereira prefers the essential oil as the best and most convenient preparation. The justness of this preference we think doubtful, for the resinous principle possesses also very active properties. On this account we have always employed the pulverized berries, although rather a bulky medicine. In the state of powder it is liable to be adulterated, chiefly with other and cheaper kinds of pepper. We have known it mixed with pulverized pimento, which may be detected by inspection with an ordinary magnifier; the particles of pimento being of a light brown, when compared with the blackish hue of the cubebs. Cubebs, when in the pulverized state, ought to be kept in a well-stopped phial, as its essential oil is very volatile, and when kept in paper, the latter absorbs a large proportion of it.

*Ergot.* Without entering into the controversy regarding the nature of ergot, we may state that it has been almost proved that it is a parasitical fungus. Mr. Pereira gives a minute account of its appearance and structure, accompanied with some woodcuts. The following observations of Mr. Pereira, which our own experience enables us to corroborate, are important:

"The ergot of rye is fed on by a little acarus, which I have called A. Ergotæ. It is about one fourth the size of the cheese-mite. This animal destroys the interior of the ergot, and leaves the grain as a mere shell. It produces much powdery excrementitious matter (Quekett). In four months, 7½ ounces of this fæcal matter of the acarus were formed in seven pounds of ergot. I have some ergot which has been kept for three years in stoppered glass vessels without being attacked by the acarus, and it has all the characteristics of good ergot. It is advisable, however, not to use ergot which has been kept for more than two years." (p. 599.)

When the decomposition of the ergot occurs, it is generally owing to the presence of moisture or imperfect desiccation; for we have seen ergot kept for several years, in a perfectly entire state, when it had been previously thoroughly dried. It should not, however, be kept in the pulverized state, as it is thus rendered more liable to absorb moisture from the atmosphere. No precise chemical means are known for testing its activity, although Mr. Pereira enumerates the effects of several reagents. We may state, however, from our own experience, that if the grains have their usual form, blackish-brown colour, peculiar farinaceo-acrid but scarcely definable taste, pinkish or pinkish-white fracture, accompanied with a peculiar odour, easily recognized, but not capable of accurate description, we have rarely known the medicine to fail in producing effects on the parturient uterus. The analysis of this agent has not added anything important to our therapeutic formulæ. Mr. Pereira states that the specific gvavity of ergot is somewhat greater than that of water; while Mr. Wright (Edin. Med. and Surg. Journ.) says that it is lighter than this fluid, and even assigns this character as one of the signs of its activity. We tried six entire grains; four of them floated in water, and two sank to the bottom, but on dividing those that floated, one half of each grain sank to the bottom while the other floated. It is obvious from this that the specific gravity is not a test of the quality of this subtance. There is one question connected with the use of ergot, which it is of vital importance to determine, and about which authors differ materially; viz., whether it proves fatal to the child when exhibited during labour.

Dr. Dunglison gives a very full and excellent account of the properties and powers of ergot, but he combats the opinions entertained by many that it is injurious to the fœtus. When this agent is employed judiciously and in the proper dose we believe that injurious results are rare; but on the other hand, when given in large doses, at improper stages of the labour, during the existence of formidable impediments or during first labours that are lingering, the danger of inducing the death of the child is very considerable. Various hypotheses have been formed by authors to account for its modus operandi in killing the child. Some have supposed that it does so by the violent continuous action of the uterus causing an obstruction of the circulation of the fœtus. This conjecture is by no means improbable, and is supported by the physiology of natural labour. In the natural process, after the occurrence of one uterine pain, there is an interval of repose before the accession of another; and any partial injury from pressure during one of these efforts is recovered from, by the fœtus, during the intervals of ease. But in labour accelerated by the ergot of rye, the uterine pains have generally no interval, but are positively continuous; and if the child be not expelled before the expiry of several hours, it is fair to infer that the constant pressure must have a tendency to cause asphyxia. As an additional proof that this explanation is correct, we have repeatedly observed, when the pains were violent and continuous, after the exhibition of ergot, or when the delivery was protracted much beyond the usual period, that the child was languid or did not respire for some minutes, which symptoms were accompanied with lividity of the lips and laborious breathing. The narcotic influence of the ergot has also been brought forward to account for the asphyxia of the fœtus; but if it operated in this manner, the child would labour under the injurious effects of the poison for some time after birth, which is by no means the case, for as soon as the function of respiration is completely established, it appears as lively as other new-born infants. Another hypothesis has been advanced, viz., the detachment of the placenta previous to the birth of the child; but if this opinion were tenable, the discharge of blood which usually occurs on the detachment of the placenta ought to take place immediately after the expulsion of the child, which is not agreeable to experience.

Besides the ordinary obstetrical rules which are laid down by authors for the exhibition of ergot; there are two points connected with its administration, which are not alluded to by Mr. Pereira, and which have not been prominently brought forward by any writer on the subject; viz., the dose that should not in ordinary cases be exceeded, and the more frequent death of the fœtus after its exhibition in first labours. The quantity that we have from experience, and we have used it frequently, found safe, is from twenty to twenty-five grains, given in the form of infusion, the pulverized ergot being also swallowed. On theoretical grounds a person might be apt to imagine that if ergot of rye were harmless in second or subsequent labours, it could not be injurious in the first. If, however, we attend to the fact, that the large majority of first labours are tedious; and that the ergot has by no means the same influence over them as it has in other cases, we shall at once see that the fœtus must be much longer under the continuous compression of the uterus in the one case than in the other. So much are we convinced of the truth of what has now been stated, that though we exhibit ergot frequently when the os uteri is sufficiently dilated, and the head well advanced, we have not for several years prescribed it in *a first case* under the same circumstances. Mr. Pereira shortly alludes to the ergot as occasionally causing rupture of the uterus, especially if there be any mechanical obstruction to the delivery, such as distortion; and without questioning the fact, that such instances may have occurred, we are satisfied that this agent has in general very little if any influence in producing this accident; for in the large majority of recorded cases of rupture it had not been exhibited. We have ourselves had opportunities of witnessing a few cases of this nature, and in none of them was this agent exhibited; indeed the cause in all of them was not ascertained; but in all, the labour was tedious, although the pains were by no means energetic. Mr. Pereira is satisfied by the evidence recorded on this point that the ergot is capable of exciting abortion or premature labour. The majority of recent writers on this subject are of this opinion, and we are inclined to agree with them; at the same time we think that farther experiments are required to determine this matter conclusively.

We have no room for any remarks upon the use of ergot in the expulsion of the placenta, in hemorrhage from the uterus both before and after delivery, in the expulsion of clots, hydatids, and polypi from the womb; in general hemorrhages, in leucorrhœa, amenorrhœa &c.; but there is ample and good instructions on these and other points to be found in the works of Pereira and Dunglison. Ergot of rye has a place in all the works under our present notice, at least in their lists of Materia Medica; and its character is referred by all but the Danish

College to a species of fungus: this college terms it *Semina Monstrosa*, which is undoubtedly a very convenient method of solving a dispute about its classification.

*Saccharated Carbonate of Iron*, (Carbonas ferri saccharatum. *Ed.*) There can be no doubt of the fact that the precipitated carbonate of iron, or what should properly be called the sexquioxide of iron, is very inferior in medicinal operation to the protocarbonate of the same metal. The protocarbonate of iron, however, when exposed to the air, speedily parts with its carbonic acid, and absorbs an additional portion of oxygen; it is therefore of considerable importance in a therapeutic point of view to prevent this change. It has been long known that saccharine and some other bodies, when united with the proto-salts of iron, retard their peroxidation, and various preparations of the protocarbonate have been devised to effect this object—which, however, they do only partially—such as the pil. ferri c., mixt. ferri c. of several of our pharmacopœias. We believe that there is not enough of sugar in either of these preparations to prevent decomposition, and the process recommended by Dr. Clarke ten years ago is greatly superior. He directs the soft precipitate to be mixed with sugar and aromatic powder, so as to form an electuary. The preparation newly introduced into the Edinburgh Pharmacopœia under the above name is calculated, we think, to effect the object referred to better than any previous process, in so much as the mixture is dried, and thereby rendered less liable to absorb oxygen. A similar preparation is to be found in the Danish Pharmacopœia, which is called *Ferrum subcarbonicum saccharatum*; but the proportions of the sulphate of iron and subcarbonate of soda have not been given. Sir James Wylie adopts the plan of washing the precipitate with honeyed water, in order to prevent the action of the air, and afterwards forms it into a kind of dense extract with honey, by the aid of the water-bath. No formula of this kind is in the London Pharmacopœia. The test of Purity of the Edinburgh College, viz., its grayish-green colour, and its solubility in muriatic acid, with brisk effervescence, will easily distinguish it from the sexquioxide of iron, which possesses neither of these properties.

*Iodide of Iron*, (Iodidum Ferri.) This preparation was introduced into practice by Dr. A. T. Thomson, and is now pretty frequently prescribed, and has been admitted into the London, Edinburgh, Russian, and other Pharmacopœias. This shows that it has got more or less of the confidence of the profession. We think it a good preparation; but we must add that its virtues have not been recorded to an extent sufficient to prove its superiority to other preparations of iodine, or to establish the peculiar advantages which result from the simultaneous action of the iodine and iron. Sir James Wylie states that it may be employed externally in the form of bath in scrofulous affections; half an ounce to an ounce being dissolved in water for this purpose.

In the Edinburgh Pharmacopœia iron wire is employed in making this preparation, which is much more pure than iron filings, the article recommended in the London and Russian Pharmacopœias; much more is directed than can be dissolved in the first of these works, but this is no great objection, as it is cheap and ensures its more ready solution, and the more complete saturation of the iodine.

*Test of Purity.* The Edinburgh College state that it is entirely soluble in water or nearly so; forming a greenish solution. Would this test distinguish it from the proto-salts of iron?—We think not. That of the London College is better, although not very complete, but the subject is still open for farther investigation. The preparation and therapeutic properties of this agent are very fully detailed by Dunglison.

*Hydrated Sesquioxide of Iron,* (Ferrugo). Recent experiments have established the discovery of MM. Bunsen and Berthold, that the hydrated peroxide of iron proves, in many instances, an antidote to the poisonous effects of arsenious acid, particularly when exhibited frequently and in large doses. A chemical union takes place between them, and in this manner an insoluble arsenite of iron having little activity is the result. The dry sesquioxide of iron having been found much less efficacious than the hydrated form, it is therefore of essential importance that this should be procured at a moment's warning. We have known instances of poisoning where this preparation could not be procured, and where the mode of preparing it was totally unknown to the practitioner, so that the ordinary sesquioxide of iron required to be substituted for it. We are therefore gratified to see a process for its preparation, both in the Edinburgh and Russian Pharmacopœias. In the last named work it is recommended, when intended to be used as an antidote to arsenic, to be preserved, of a jelly-like consistence, in close vessels, the "Hydras Sesquioxydi Ferri, ut antidotum acidi arsenicosi adhibendus, humidus et nequaquam siccatus, gelatinæ quasi speciem referens, in vasis probe clausis servetur." (p. 529.) As this is a very important formula, and one which ought to be known to every practitioner, we shall quote the direction for its preparation by the Edinburgh College:

"Take of Sulphate of Iron, four ounces;
Sulphuric Acid (commercial) three fluidrachms and a half;
Nitric Acid (D. 1380), nine fluidrachms;
Stronger Aqua Ammoniæ three fluidounces and a half;
Water, two pints;

Dissolve the sulphate in the water, add the sulphuric acid, and boil the solution; add then the nitric acid in small portions, boiling the liquid for a minute or two after each addition, until it acquires a yellowish-brown colour and yields a precipitate of the same colour with ammonia. Filter; allow the liquid to cool; and add in a full stream the aqua ammoniæ, stirring the mixture briskly. Collect the precipitate on a calico filter; wash it with water till the washings cease to precipitate with nitrate of baryta; squeeze out the water as much as possible; and dry the precipitate at a temperature not exceeding 180°. When this preparation is kept as an antidote for poisoning with arsenic, it is preferable to preserve it in the moist state, after being simply squeezed." (p. 101.)

*Iodide of Mercury,* (Hydrargyri iodidum.) This preparation is commonly called the proto-iodide of mercury to distinguish it from the deuto-iodide of the same metal. Their appearance and properties are very different; yet they have sometimes been confounded, or at least considered so nearly resembling one another in physiological qualities as to be prescribed in the same doses. For example, Dr. A. T. Thomson, in his Materia Medica (vol. i. p. 342), prescribes them in the same dose, and states that "for external application an ointment is prepared with half a drachm of either of the iodides and one ounce of lard." Dr. Dunglison, however, has given correct information upon this point, and he states that the deuto-iodide resembles corrosive sublimate, while the proto-iodide resembles calomel. He also quotes Rayer, who considers the deuto-iodide more active than corrosive sublimate. The iodide is certainly an irritant, when exhibited in large doses, and proves poisonous to animals, but it may be exhibited, to the extent of two or three grains, when quite free from the biniodide, without causing any particular excitement in the system, except sometimes a laxative effect. It has been recommended strongly by Lugol in the form of ointment to scrofulous swellings, in the proportion of twenty to forty grains to an ounce of lard, and is occasionally of use, but like all the other substances which are applied by friction to swollen glands, it often does as much harm as good, by exciting a tendency to suppuration. As an application to ulcers, however, and some cutaneous diseases, it is sometimes beneficial. It acts as a mild mercurial when given internally, and we have often exhibited it in syphilis and diseases of the liver, occurring in scrofulous constitutions, in the dose of a grain night and morning, salivation being produced in eight or ten days. The Edinburgh College have not thought proper to insert this medicine into their Pharmacopœia; it has been admitted into all the other works on our list.

*Test of Purity.* The test of the London Pharmacopœia is not precise in its application, with the exception of its insolubility in chloride of sodium. Mr. Pereira states that

> "It is a greenish-yellow powder, whose sp. gr. is 7·75. It is insoluble in water, alcohol, or an aqueous solution of chloride of sodium; but is soluble in ether, and slightly so in an aqueous solution of iodide of potassium. When heated quickly, it fuses and sublimes in red crystals, which subsequently become yellow. Solar light decomposes it, and changes its colour. Heated with potash, it yields iodide of potassium and reguline mercury." (p. 481.)

To this may be added the disengagement of iodine when heated with concentrated sulphuric acid in a glass tube.

*Biniodide of Mercury*, (Hydrargyri biniodidum.) The red iodide of mercury is an extremely acrid and poisonous substance, and when applied to the skin produces vesication and excoriation. It is rarely exhibited internally, as it does not seem to possess any peculiar advantages as a mercurial, and the proportion of iodine which a minute dose contains holds out the prospect of little benefit from it, as far as its operation is concerned. The remarks of Sir James Wylie and Mr. Pereira upon its employment are very short, and they do not indicate much practical experience of its external operation. In the Russian Pharmacopœia, the proportions given for the formation of the ointment are, biniod. hydrarg. ʒss, ceræ flavæ ʒij, adip. suill. ʒvj, or 30 grs. to an ounce; and Mr. Pereira considers the following a dilute ointment in opacity of the cornea, viz. biniod. hydrarg. gr. ij, cerat. ℈ij, ol. amygd. ℈j, Now we venture to assert that the irritation produced by either of these ointments would be such that either high inflammation or intense pain would prevent the use of them for any length of time. Indeed we have often found the proportion of 10 grains to an ounce of cerate, which is recommended by several foreign authorities, in general too great, causing frequently intense pain and vesication around the margins of the ulcer. In most cases 2 or 3 grains to an ounce of cerate have appeared to us sufficient, and when the proportion is 10 grains and upwards, the ointment ought to be heated and applied with a hair pencil to prevent its action on the surrounding parts. With attention to these points, we have found this agent most beneficial in the cure of old ulcers, and lupoid and syphilitic sores, more particularly when occasionally alternated with the employment of nitrate of the silver. The ointment of the London College, viz. biniodide of mercury ℥j, white wax ℥ij, lard ℥vj, is enormously disproportioned, and is made of the same strength as that of the iodide. The Edinburgh College, perhaps considering this a debateable subject, have given no formula for its preparation. Sir James Wylie recommends 10 grains to an ounce of axunge in his history of this agent, but 30 grains are ordered in the formula.

*Preparation.* The old plan adopted in the preparation of the iodides of mercury was by double decomposition of a mercurial salt by the hydriodate of potass, and the Danish College still adhere to it; but in the London, Edinburgh, and Russian Pharmacopœias, the direct combination of the iodine and mercury by trituration is adopted, according to the plan originally proposed, we believe, by M. Berthemot.

This is certainly the most simple formula, and we doubt not answers the purpose very well, although the iodine is liable to be volatilized during the trituration. The biniodide may also be prepared very purely from corrosive sublimate and the iodide of zinc in the proportion of 100 of the former to 85 of the latter. The Edinburgh College, however, have adopted an expedient which is well calculated to ensure its purity, viz. to dissolve it in a concentrated solution of chloride of sodium, which will free it from any iodide; and the iodide itself being insoluble in that fluid might be freed from any adhering biniodide, if the same plan were resorted to. There is an objection to this solution in the chloride of sodium, that a portion of the biniodide may remain dissolved and thus be lost; besides, the proportions of the common salt and water ought to have been given, for the term concentrated is rather indefinite. For full information respecting the iodides of mercury we refer our readers to Dr. Dunglison's articles.

*Tests.* The Edinburgh College recognize its purity by its entire volatilization, its complete solubility in a concentrated solution of common salt at 212°, and its subsequent deposition in the form of fine red crystals. We believe that these tests are capable of recognizing all ordinary adulterations or impurities. Those in the London Pharmacopœia, however, are more numerous, although the mode of operating with the muriate of soda is not described.

*Indigo,* (Indicum.) The qualities of this substance as a pigment are well known in the arts, but as a medicine it has scarcely been tried by British practitioners. It has of late, however, been employed successfully on the continent in several diseases, particulary in epilepsy; and although we by no means pledge our belief to all that has been written regarding its therapeutic powers, it is fairly entitled to be tried, more particularly as this disease must in many cases be set down as incurable, and as no deleterious consequences followed the use of the agent. We find it well described in the works of Mr. Pereira, Dr. Dunglison, and Sir James Wylie, and shortly in the list of materia medica of the Danish College. Sir James Wylie speaks cautiously regarding its virtues.

"In the deficiency of proper experiments," he says, "I am not unmindful that the utility of indigo is allowed by Rhül and Dr. Spasky, being commended for the cure of certain neuroses; wherefore I have reckoned it worth while to insert this new remedy into this *small* work, namely, that it may be subjected to ulterior experiments." (p. 198.)

Many species of indigoferæ are cultivated for the manufacture of indigo. These plants, according to Sir James Wylie, grow in Java, India, Arabia, Egypt, the Philippine Islands, Luconia, in the northern and southern provinces of Hindostan; and are also very frequently met with in Tinnevelli, Gautimala, and Caraccas. When in flower they are cut with sickles; and being arranged in strata in proper vessels, water is poured upon them. The liquor runs into fermentation, being green at the beginning, but changing into a coppery red colour. Lime-water is added to it and mixed by agitation, it is then withdrawn by the inferior orifice into another vessel and allowed to settle, and afterwards deposit the pigment in the form of purple flakes. Commercial indigo consists of indigo-blue (indigotin) which is volatilized at 550°, indigo-brown, indigo-red, and a glutinous substance. Berzelius states that indigo seldom contains the half of its weight of blue colouring matter, more frequently less, that it is adulterated with sand, powdered bricks, and similar substances, more rarely with starch. Good indigo should float on the surface of water, and the less its sp. gr. the better is the quality. It ought to have a deep blue colour, and the more brilliant and analogous to copper the colour becomes by friction, the purer is the indigo.

The physiological effects of indigo in full doses are chiefly nausea, vomiting, and diarrhœa, colic pains, and other dyspeptic symptoms. The urine has generally a dark-brown violet colour. It ought to be commenced in the dose of a few grains, exhibited three or four times a day, which is to be gradually increased to drachms; so that the patient may take one or two ounces in twenty-four hours. On account of its lightness when pulverized it should be given in the form of electuary.

*Arrow-root.* (From Maranta Arundinacea.) We are induced to notice this article here, although now so well known as an article of diet, on account of the various opinions held respecting its nutritive properties, when compared with the amylaceous products of Great Britain. Mr. Pereira states that the maranta arundinacea was brought from the island of Dominica to Barbadoes, and from thence it was sent to Jamaica. The native Indians, according to Colonel James Walker, apply the bruised root to the wounds which they receive by poisoned arrows. In Jamaica this plant is cultivated in gardens and provision grounds. The following is Mr. Pereira's account of the extraction and properties of the fecula:

"The roots (tubers), when a year old, are dug up, well washed in water, and then beaten in large, deep, wooden mortars to a pulp. This is thrown into a large tub of clean water. The whole is then well stirred, and the fibrous part wrung out by the hands and thrown away. The milky liquor being passed through a hair-sieve, or coarse cloth, is suffered to settle, and the clear water is drained off. At the bottom of the vessel is a white mass, which is again mixed with clean water and drained; lastly, the mass is dried on sheets in the sun, and is pure starch. The fecula (fæcula marantæ), called in the shops West Indian arrow-root, is white, odourless, and tasteless. It is in the form either of a light opaque white powder, or of small pulverulent masses. When pressed between the fingers it feels firm, and when rubbed, produces a slight crackling noise. Examined by the microscope it is found to consist of granules, which are never perfectly spherical, but are frequently elliptical, sometimes obscurely triangular, and occasionally have the shape of a painter's muller." (p. 679.)

*Purity.* "Potato-starch (sold in the shops as English arrow-root) is said to be sometimes substituted for the Indian arrow-root. The fraud may be readily detected by a good microscope. The particles of potato-starch are distinguished from those of Indian arrow-root by their larger size, and by the concentric markings on their surface.

"Recently a fecula has appeared in the shops, under the name of Tous les Mois, or Starch of the Canna coccinea. It comes from St. Kitt's; and is said to be prepared by a tedious and troublesome process, from the root (rhizome) of the plant above named. It has been recommended as forming a valuable article of food for the invalid. On a microscopic examination of it, I find that its particles agree in form and structure with those of the ordinary potato-starch, from which, however, they are distinguished by their larger size." (p. 680.)

Even by the aid of an ordinary magnifier, such as is used in the examination of plants, we have been able to detect the difference in appearance that exists between some of the varieties of feculæ. Viewed through this medium, potato-starch presents the appearance as if densly studded with small globules of mercury; arrow-root has the aspect of pulverized and rather imperfectly-crystallized sugar, and wheat-starch only exhibits a glistening appearance.

The sophistication of arrow-root with potato-starch is not injurious as far as the health of the public is concerned; for though the former is the strongest fecula, and forms the most consistent jelly, yet the difference between them in point of digestibility and nutritive power is very immaterial; at the same time, as the one is frequently quadruple the price of the other, it cannot be considered in any other light than as a fraudulent imposition.

East Indian arrow-root is procured from the curcuma angustifolia, and it is sometimes mixed with that prepared in the West Indies, although generally bought by starch-makers. Mr. Pereira states that it may be distinguished by its want of firmness and crepitation, when pressed or rubbed between the fingers, and that it resembles finely pulverized salt, or bicarbonate of soda. Brazilian arrow-root is the fecula of the iatropha manihot.

*Milk*, (Lac vaccinum.) According to Pereira, the sp. gr. of milk is about 1·030, when recent it is slightly alkaline, and when viewed by the microscope it is observed to consist of myriads of globular particles floating in a serous fluid. These globules consist essentially of butter, and disappear on the addition of caustic alkali.

The following is the analysis of cow's milk, according to MM. O. Henry and Chevallier:

| | |
|---|---|
| " Caseum | 4·48 |
| Butter | 3·13 |
| Sugar of milk | 4·77 |
| Various salts | 0·60 |
| Water | 87·02 |
| Total | 100·00 |
| Solid substances | 12·98" (p. 1405.) |

The characteristics of good milk, as given by Pereira, are as follow:

"The following are the essential morbid changes which have been recognized in milk: Want of homogeneousness, imperfect mobility or liquidity, capability of becoming thick or viscid on the addition of ammonia, and presenting, when examined by the microscope, certain globules (agglutinated, tuberculated, or mulbery-like, mucous or pus globules) not found in healthy milk. Hence, then, good milk should be quite liquid and homogeneous, not viscid; and should contain only spherical transparent globules, soluble in alkalies and ether; should not become thick when mixed with ammonia; and should form a flocculent precipitate with acetic acid, but not be coagulated by heat. The relative quantity of cream afforded by milk is estimated by a graduated glass tube, called a lactometer." (p. 1406.)

Mr. Pereira does not take any notice of the adulteration of milk, so commonly practised. When flour and water are used for this purpose, the former is easily detected by the blue colour it strikes with iodine, particularly if the curd be previously separated by sulphuric acid. In Paris, milk has been sophisticated by an emulsion of almonds or of hemp seed sweetened with sugar. This factitious milk may be detected by the oily nature of its curd. When the latter is pressed between the fingers or on paper the oil exudes from it, which is not the case with the curd of pure milk. The quantity of curd procurable from milk so adulterated is always greatly diminished. When milk during summer curdles or coagulates by the formation of lactic acid, the fluidity is restored by the addition of a small quantity of subcarbonate of potash. Some very excellent observations are made by Pereira on the influence of many medicines taken by the mother over the suckling infant, and the care requisite in the selection of a nurse. From observation, we are quite satisfied of the truth of this author's remarks: we have often witnessed the injurious effects of the milk furnished by dyspeptic nurses, particularly when they were addicted to the use of ardent spirits.

Our limits do not permit us to extend further our notices of individual formulæ and remedies. We shall therefore conclude the article with a few general remarks on the character of the works before us, at the same time adverting briefly to a few particulars which could not be properly introduced before.

*Edinburgh Pharmacopœia.* In the present edition of this work, a great many improvements have been adopted, by the addition of approved therapeutic agents, the introduction of tests, the alteration of some of the formulæ, and in the expulsion of several inert or inefficient substances from the list of medicines. The substitution of measures instead of weights for ascertaining the quantities of fluids, as previously adopted by the London College, will facilitate very much the compounding and correct administration of medicines. The additions, which are considerable, are for the most part very judicious, almost all the new medicines being generally acknowledged to be useful agents. The number of articles expunged seem to be nearly as numerous as those introduced, and the exclusion of almost all of them is perfectly proper. We think, however, that the Artemisia Santonica which is a good vermifuge, Mace which is useful in checking vomiting, particularly in children, Oleum Lauri nobilis, which is an excellent rubefacient, ought to have been retained. Why Anchusa, which is used for colouring oils, tinctures, &c., has been expunged, and Papaver rhæas introduced for a similar reason, is not very obvious.

The tests employed for ascertaining the purity of medicinal agents are in general very precise, capable of detecting the usual adulterations, and a considerable number of them are more definite than those adopted by the London College, which in many instances consist chiefly of the general chemical characteristics of the substances. Some of those, however, of the Edinburgh College would have been rendered more complete if their prominent chemical characteristics had been added; for as those who sophisticate medicines are constantly on the alert to avoid discoveries, some new method might be resorted to by them to avoid detection. The Edinburgh College have the merit of introducing a number of very valuable tests for various vegetable substances, which are by far the most difficult of application, and some of them are exceedingly good, such as that for the sulphate of quina (quinometry,) which Mr. Pereira considers the best method. The mode of ascertaining the quantity of morphia in opium is also good (morphiometry), but, as Mr. Pereira remarks, it will require some care in the application of the heat, as this alkaloid is soluble in a solution of subcarbonate of soda. The test for olive oil is that discovered by M. Poutet, and it is very precise, for no other fixed oil subjected to the acid nitrate of mercury solidifies so rapidly or acquires in so short a time the same hardness of texture. It is capable of detecting a very small proportion of poppy or linseed oil; but the presence of the latter, if not cold-drawn, may also be discovered by the yellowish colour it communicates to strong alcohol. In this country rape oil is most frequently employed for its sophistication, and a small portion of it may be mixed with olive oil without preventing the solidification; but the resulting compound with the acid nitrate of mercury is always softer and more brown in colour, and these characteristics are much more distinct when the quantity of rape oil is much increased. We are surprised that this test for olive oil is not in the London, Danish, or Sir James Wylie's Pharmacopœias. We admire the simplicity of the Edinburgh test for arsenic, which we have always found sufficient, viz., complete sublimation, for we should think there is no volatile substance cheap enough to be employed as an adulterating ingredient. Their test for vegetable matter or essential oil in alcohol, viz., the nitrate of silver, is elegant, and it might also detect some of the usual substances employed in its rectification. Alum is stated not to be subject to adulteration, and we believe that this is by no means common; but Mr. Pereira mentions that it is sometimes mixed with the ferro-sulphate of potash, and that it cannot be distinguished from the latter by its form, colour, or taste, but is readily detected by potash, which throws down oxide of iron, and by tincture of nut-galls which communicate a bluish-black colour to it. The test for camphor is very simple and good, viz., its complete sublimation. The mode of recognizing the powder of conium by trituration with aq. potassæ is calculated to be very useful; but even simple friction of the leaves eliminates the odour of this narcotic. The test for oil of cinnamon is its conversion nearly into a crystalline mass by nitric acid. Will this distinguish it from oil of cassia? Mr. Pereira states that oil of cassia is nearly solidified with nitric acid. Although, however, it may not be capable of distinguishing the adulteration of oil of cinnamon with oil of cassia, we believe it to be an excellent test for recognizing the presence of many other oils, such as oil of turpentine, fixed oils, &c. An intense action follows the admixture of nitric acid with oil of sassafras, and when the latter is combined with oil of cinnamon its solidification is prevented. This experiment we tried, because we thought it likely that oil of sassafras, as belonging to the same natural family with oil of cinnamon might behave in a similar manner with nitric acid. Even oil of olives, although solidified by the acid nitrate of mercury when mixed with oil of cinnamon, also prevents its complete solidification.

Acetate of potass is stated not to be subject to adulteration; but if it be prepared from an impure carbonate of potass it must contain impurities. The tests for this salt in the London Pharmacopœia are pretty good. The mode employed for ascertaining the purity of bitartrate of potass is greatly superior to that in the London Pharmacopœia. The ordinary and pretty well known test for castor oil, viz., its solubility in strong alcohol, has been introduced, and we are astonished that it is not in the London Pharmacopœia.

The tests for the adulteration of scammony are of considerable value, for its high price offers great inducements to sophisticators.

Argel leaves are directed to be removed from Alexandrian senna by picking, but their characters are not given, which is an omission. Pereira gives the following account of argel leaves:

"The argel plants are collected by the Arabs, in the valleys of the desert to the east and south of Assouan. The *leaves* found in Alexandrian senna are distinguished from the senna leaflets by their being equal-sided, by the absence or imperfect development of the lateral nerves, by their paler colour, thicker and more coriaceous texture, by a yellowish exudation frequently found on them, and generally, though not invariably, by their greater length. By careful picking the flowers may be detected: they are white, and in small corymbs. The fruit, as found in Alexandrian senna, seldom exceeds in size that of a good-sized orange-pip. It is an ovoid follicle, tapering superiorly, brown, shrivelled, and contains several seeds." (p. 1165.)

The test with iodine for the adulteration of mustard with flour is very properly introduced, as this sophistication is frequently practised. The test for strychnia is more definite than that in the London Pharmacopœia, but its characters are well described by Mr. Phillips.

We shall now notice shortly one or two of the processes, which have been much improved. The improved process for the preparation of tartaric acid is literally adopted from that in the London Pharmacopœia, as well as that for citric acid ; the latter, however, has some modifications which ensure the complete saturation of the sulphuric acid employed. The Edinburgh college have also adopted the method of preparing benzoic acid by sublimation, which is the most convenient and productive. Their process for muriate of morphia is undoubtedly the best, and we believe that it has been practised on the large scale with a productive return. That for sulphate of quina is also good and must be cheap, from there being no alcohol employed. We also highly approve of the introduction of the acetate of morphia ; for though the muriate is more generally employed, the acetate is at least equally good. We have often practised a cheap method of preparing it from laudanum, by double decomposition with acidulated acetate of lead, the acetic acid being added to prevent any precipitation of the morphia with the resulting meconate of lead. The acetum opii, which is now introduced from the Dublin Pharmacopœia cannot be considered a proper acetate of morphia, although it is stated to produce similar effects to the salts of morphia. The Edinburgh college, we think, have displayed good sense in delaying a process for bromide of potassium, until its powers have been more thoroughly tested, even although it has been introduced into the London Pharmacopœia. The Edinburgh college state that distilled waters may be prepared by agitating the essential oil of the plant with water. This plan, we apprehend, will not impregnate the water sufficiently; and most practical pharmacians, who adopt this method, triturate the oil with sugar and a little water in a mortar, before the whole of the latter is added. The introduction of enemata are calculated to be useful. Starch is directed for the preparation of this opiate enema, but a simple solution of laudanum in tepid water in general answers equally well.

The number of extracts have been greatly increased, and most of them are valuable. The new method of making the tinct. mur. ferri from the red in place of the black oxide of iron, as formerly adopted, is an improvement, for the tincture of the proto-muriate always ultimately becomes peroxidised, and it is besides often deficient in strength. The formula for calomel has been improved, and several mercurial preparations of little practical value have been expunged. The iodide of lead is new; it is prepared from the nitrate of lead, which is superior to the process of the London college from the acetate, in as far as there is no risk of the formation of a sub-iodide, which sometimes occurs, particularly when the acetate of lead is not acidulated with acetic acid, as has been shown by M. Denot.

The *mistura scammonii* is a convenient formula for children: it is as follows:

"*Mistura Scammonii.* Take of resin of scammony seven grains, unskimmed milk three fluid ounces: triturate the resin with a little of the milk, and gradually with the rest of it till a uniform emulsion is formed." (p. 119.)

Several new and important formulæ for pills have been added; and it would have been an improvement if the soap employed in the manufacture of some of them had been directed in powder, as in the Danish Pharmacopœia, for we are persuaded that this article is often very imperfectly combined with the other ingredients. Syrupus rhamni, which is a convenient and pretty certain purgative for children, and the improved syrupus sennæ, are valuable additions; we give the formula for the latter:

"*Syrupus Sennæ.* Take of senna, four ounces; boiling water, one pint and four fluidounces; treacle, forty-eight ounces; infuse the senna in the water for twelve hours; strain and express strongly through calico, so as to obtain a pint and two fluidounces at least of liquid. Concentrate the treacle in the vapour-bath as far as possible, or till a little taken out upon a rod becomes nearly concrete on cooling; and while the treacle is still hot, add the infusion, stirring carefully, and removing the vessel from the vapour-bath as soon as the mixture is complete." (p. 154.)

In the preparation of tinctures, percolation is recommended in the majority of instances. Several new tinctures have been added, and there is a modification in the preparation of laudanum, by which the opium is submitted first to the action of hot water, and then to that of rectified spirit.

We are surprised that the Edinburgh college, among many valuable additions, have not added to their list the iodide of sulphur, which is a valuable remedy for lupus, porrigo and lepra; but neither is it to be found in the other works noticed in this article, with the exception of a short account of it by Mr. Pereira and by Dr. Dunglison. The present edition of the Edinburgh Pharmacopœia has been published in a much more portable and convenient form than the last, and though the type is smaller, yet it is sufficiently distinct and legible. It is on the whole a work of great merit, indicating accurate and extensive knowledge of similar and other scientific treatises; and is, both theoretically and practically, at least equal to any similar pharmacopœia.

*Pereira's Materia Medica.* On a former occasion (vol. VIII. p. 353) we noticed the first volume of this work. The second volume treats of vegetable and animal pharmacology. The author adopts a natural-historical in place of a physiological or therapeutical classification, a plan which was many years ago adopted by the late Dr. Duncan of Edinburgh, and which, we believe, is still followed by his distinguished successor Dr. Christison. This plan of teaching and writing is undoubtedly more in accordance with abstract science than the mode generally adopted of describing the different substances under a classification derived from their action on the healthy or diseased functions of the body. At the same time it must be admitted, that the latter methods have some advantages in a practical point of view, in as far as such an association fixes in the student's memory more quickly the peculiar action of the agent described. The tabular view which is given of the history and literature of materia medica must be very useful to those engaged in such investigations, including as it does a list of the works ancient and modern, which have been published in almost every country in the world. We consider Mr. Pereira's descriptions of the vegetable and animal materia medica as exceedingly clear, methodical, and minute, including an account of many substances not generally described by writers on pharmacology. In noticing the first he gives the history, botany, habitude, description, commerce, composition, chemical characteristics, physiological effects on the brain, spinal system, digestive system, vascular system, respiratory system, urinary system, sexual system, cutaneous system, topical effects, uses, administration, preparations. We refer our readers to the article opium, which occupies about forty-eight of his large and closely printed pages, to show the admirable minute and methodical manner in which this subject is handled; comprising, in short, every thing connected with its history and properties that could be desired by the practitioner, or even wanted in ordinary juridical investigations. Several of his other articles are equally elaborate.

The second volume fully supports the character which we expressed of the first. We have no hesitation in stating that, in almost every particular, it is superior to any similar work in the English language; and we know of none which exemplifies so completely the rare combination of minute knowledge in natural history, botany, and chemistry, with sound and practical therapeutics. We fear, however, although we would regret this, that the minuteness of detail, as well as its price, will limit to a certain extent its general employment as a class-book for students. We suspect that the price has been unnecessarily increased by the introduction of wood-engravings, some of which are not necessarily connected with the subject, and many do not convey a good idea of the substance intended to be represented.

*Sir James Wylie's Military Pharmacopœia.* This work may be considered both as a treatise on pharmacology and a pharmacopœia, and in many respects it is similar to our British dispensatories. He divides it into three parts: in the first he treats of vegetable and animal medicines; in the second, of mineral and chemical medicines; and in the third, of preparations and formulæ. The work is commenced by a short exposition of the modern chemical nomenclature, with an account of the medical weights and measures, which appear to be the same as those adopted in this country. The medical pound contains 5760 grains, while the Russian mercantile pound contains 6586·08. The same liquid measures are also employed; namely, the gallon, pint, fluid ounce, fluid drachm, and minim.

The descriptions in the pharmacological part of the work are in general clear and accurate, and comprehend the modern discoveries in natural history, botany, and chemistry. Short accounts are also given of some dietetic articles, as the gramineæ, the sheep, ox, &c. The toxicology of many articles of the materia medica is pretty fully described; and there is a long and excellent article on arsenic, accompanied with a comparative table of precipitates from arsenical and other solutions by the various reagents employed as tests for this metal. Tables of the contents of various Russian and several other mineral waters are also given. Almost all the new remedies that have lately flowed in upon us so copiously have been introduced; and there are formulæ for the preparation of Carlsbad and Cheltenham salts, a bath of the sea containing iodine and bromine, &c. Even the mode of manufacturing the capsules of copaiva is described, which the author calls *baccæ.* We do not quarrel with him for describing these and many other substances in his pharmacology; but when particular formulæ are given for their preparation, in a national pharmacopœia, this stamps them with an authority which should only be conferred when their powers have been fully ascertained. A very great number of inert vegetable preparations have been admitted, and there are no fewer than forty-four formulæ for extracts, among which are those from fumaria officinalis, triticum repens, marrubium vulgare, matricaria chamomilla, saponaria officinalis, tanacetum vulgare, achillea millefolium, inula helenium, viola tricolor, ligusticum levisticum. The extract of milfoil is said to be useful in hypochondriasis, spasms, inflation, and to those who are vexed with nocturnal pollutions. The formula for the preparation of emplastrum lithargyri, which is directed to be made with five parts of litharge and nine parts of axunge, is greatly superior to the plan recommended in the London, Edinburgh, and other Pharmacopœias, namely, with olive oil, inasmuch as the plaster is less liable to crack when spread upon calico; and we believe that many practical pharmacians use at least a third of axunge in the composition of their litharge plaster. A tincture of strychnia has been introduced, namely, strychnia 3 grains, purest alcohol 1 ounce, which is exhibited in the dose of from six to twenty drops. This will be found a useful formula for graduating correctly the dose of this dangerous medicine. The formula for the *lapis divinus*, which is occasionally used in the form of collyrium in different diseases of the eye, and recommended by some writers, is the following:

"℞. Sulphatis cupri,
Supersulphatis aluminæ et potassæ,
Nitratis potassæ, singulorum unciam;
misce, et liquefac calore auctiori; sub finem adde:
Camphoræ tritæ drachmam dimidiam. Misce." (p. 783.)

The *pilulæ colocynthidis ferrosæ* are somewhat singular in their composition; but they are stated to be useful as resolvents, aperients, cathartics, stimulants to the viscera and intestines, promoting secretion and quickening the flow of blood in the portal veins.

"℞. Extracti colocynthidis compositi drachmas tres,
Assæ fœtidæ,
Saponis oleacei, vel
Bilis taurinæ spissatæ,
Hydrochloratis ammoniæ ferrosi,
Extracti chelidonii, singulorum drachmam,
Tartratis stibii et potassæ grana novem,
Olei chamomillæ guttas triginta; si opus,
Syrupi simplicis quantum sufficit.
Misce, fiat lege artis massa, in pilulas pondere granorum duorum dividenda, pulvere lycopodii conspergendas." (pp. 749-50.)

Lugol's solution of iodine has been adopted, which is a very convenient formula, and superior to the tincture of that substance; there is also an elegant acid solution of sulphate of quina in the tincture of aurantium. The pilulæ hydrargyri are made with mercurial ointment. The decoctum cinchonæ acidulatum is a good preparation, and must contain more quinine and cinchonine than that prepared by water.

"℞. Cinchonæ cordifoliæ contusæ unciam,
Aquæ communis uncias sedecim,
Acidi sulphurici diluti drachmam.
Coque in vase leviter clauso per sextam horæ partem, et liquorem adhuc calentem per linteum rarum et vetustum exprimendo cola." (p. 698.)

In conclusion we may remark, that the military pharmacopœia has few defects when compared with its merits; and we fully credit the distinguished author when he states in his preface, that the composition of it occupied him for three years. It is throughout characterized by accuracy of research, generally by sound therapeutic views, and by an acquaintance with the modern discoveries in medical science. The Latinity is on the whole good; the work is beautifully got up, and extends to upwards of 800 pages; the type is large and distinct.

*Danish Pharmacopœia.* This new edition of the Danish Pharmacopœia was published last year, and certainly it was the incumbent duty of the college to bestir themselves after a lapse of thirty-seven years, the previous one being published in 1805. This, however, might have been overlooked (as royal colleges often possess a kind of vis inertiæ in the execution of such matters), if the work had been well managed after so much time allowed for experiment and the collection of facts. Some of the modern improvements and discoveries in pharmacy and chemistry have been introduced, but upon the whole it is decidedly deficient in these particulars; and, besides, there are retained in the materia medica, and among the formulæ, substances and compounds that have long ago been considered, at least in this country, inert or inefficacious in the cure of diseases.

Some of the tests for ascertaining the purity of the different substances are pretty good, but many of them are defective. The Danish college have not availed themselves to a proper extent of the recent researches and publications connected with this subject. It is stated at page 9, under the article gold, that "solummodo in aqua regia solubile." Now this metal is also dissolved by chlorine, bromine, &c. Sulphate of copper (Blaa vitriol) at page 22, is said to be soluble in two parts of water. This is true as regards boiling water, but it requires four parts of water at 60 degrees for its solution. The presence of copper in extracts is directed to be ascertained by the old and indefinite method of thrusting a polished plate of steel into them. The tests for the hydriodate of potass are simply its form, the proportions of its solubility in water and in alcohol. After the formula for the carbonate of magnesia (magnesia carbonica, kulsyret magnesia), it is added,

> "Sit massa nivea, voluminosa, acido sulphurico diluto prorsus solubilis, quæ solutio neque kali carbonico neque acido oxalico turbetur." (p. 184.)

This is an obvious error. The very process for its preparation shows, that when magnesia is dissolved in diluted sulphuric acid, it will be rendered turbid by a carbonated alkali. The test for the presence of oil of turpentine, or ætherial oil in oleum petræ (petroleum), is fuming nitric or sulphuric acid, which calorifies, colours, or thickens the sophisticated oil. Under nitric acid, p. 90, after nitrate of barytes and nitrate of silver are mentioned as reagents, the following indefinite expression is employed: "affusa aqua hydrothionica non prodat metalla." And here we may state, that the latinity of this work is even less classical than usual in such compositions. The Danish Pharmacopœia, however, contains almost the whole of our old and most efficient medicines. The authors of it have also introduced a considerable number of the "new remedies," such as hydrocyanic acid, croton oil, iodine, iodides of mercury, morphia, strychnia, veratria, acetic ether, muriate of gold, sulphuret of carbon, phosphorated ether, dry phosphoric acid, ergot: this evinces a desire in the college to adopt modern improvements, although several of them might have with propriety been omitted.

The chemical formulæ are in general tolerably correct, although some of them are objectionable. For example, calomel is prepared by precipitation from the protonitrate of mercury, and for the latter there is also a separate formula. In the preparation of the sulphuret of potassium, the proportions of sulphur are too great, namely, 7 parts of kali carbonicum and 4 of sulphur. In preparing morphia, the expensive process by alcohol and ether to remove the narcotine is adopted. There are processes for purifying sulphuric, succinic, and nitric acids; that for the latter is accomplished by the nitrate of silver, which might be effected nearly as well and more cheaply by purifying the nitrate of potass previously to distillation, as directed in the Edinburgh Pharmacopœia. The process for sulphate of quina is complicated and expensive, muriatic acid, caustic alkali, lime, and spirit of wine, being all employed. There is also one for the muriate of quina, by the decomposition of the sulphate with muriate of barytes. The process for liquor ferri muriatici is objectionable, inasmuch as the peroxide of iron is directed to be boiled with muriatic acid, which will volatilize the latter. Maceration would have answered the purpose sufficiently, as directed in other pharmacopœias. The vinum colchici is made from the seeds, in the proportion of two ounces of them to one pound of white wine, and in our opinion is a more certain preparation than that made from the root, as in the London and Edinburgh Pharmacopœias. The tinctura cantharidis concentrata would form, we doubt not, a very good vesicant, being directed to be made with 12 ounces of most rectified spirit of wine and 3 ounces of cantharides. The spirit of wine however has only a sp. gr. of 0·835 to 0·845, notwithstanding the verbum sesquipedale with which it is accompanied, namely, "rectificatissimus." There is a process for the combination of Irish moss with chocolate, which we shall quote, as it may prove interesting as a dietetic prescription.

"Pasta cacao cum lichene carragheno. Chocolade med carraghen-mos.

℞. Seminum cacao modo nuper descripto in massam subtilissimam redactorum,

Sacchari albi pulverati, singulorum libras duas,

Lichenis carragheni pulverati uncias tres.

Quæ bene commixta solito modo in taleolas quadrangulatas formentur." (p. 204.)

The pulvis jalapæ comp. is made with 2 parts of jalap and 1 of sulphate of potass. At p. 124 there is a regular confectionary process for encrusting the seeds of the artemisia santonica with sugar, in the same way as coriander and caraway seeds are so manufactured. We doubt very much, that even a thick stratum of sugar will so conceal the bitter taste of this vermifuge, as to render it agreeable to the gustatory organs of children. There are several decoctions of bark, and the decoctum chinæ acidum is made by adding aromatic sulphuric acid to the ordinary decoction. It would have rendered the preparation more effective, if the acid had been added to the water before the boiling. The emplastrum stibiatum is not a good formula, being made by adding 1 part of tartar emetic to 3 parts of adhesive plaster, as there is some risk of decomposition. It is better, when this salt is employed in the form of plaster, to dust it upon a piece of adhesive strap. The extractum ferri pomatum is made by maceration, and subsequent boiling of iron filings with apple-juice, which we doubt not will form a very good preparation of iron. There is a singular formula, inserted at p. 155, named farina hordei preparata (bryst-meel—breast-meal.) This preparation is directed to be made by putting barley flour into a sack, and boiling it in water for fourteen hours in a copper cauldron; then taking it out and removing its grey exterior, dividing the remainder into slices, which are to be dried, pulverized, and put into close vessels, in a dry place. What advantages this process can have we cannot conceive.

In the Danish Pharmacopœia, the weights employed are those commonly called the apothecaries'. The standard liquid measure ("mensura nostra Dan. Pol.") is equal to thirty-two ounces. Some excellent rules are given for the dispensing of medicines, which if rigidly enforced, as we doubt not they may be in Denmark, will prevent apothecaries from adding anything to pills, mixtures, &c., to facilitate their manipulation, or to render them more agreeable. The normal weight of the pill (unless otherwise prescribed) is two grains, and the purified medicine must always be furnished if not otherwise specified. A table of the doses of *heroic* medicines is given, which cannot be exceeded without special prescription by the physician. The old names are, generally speaking, retained, but are accompanied, in the department of materia medica, with an account of the chemical composition of the agent, or with the botanical name of the vegetable from which it is procured, as well as with short descriptions of the substances themselves; and upon the whole this part of the work is pretty well executed.

*Phillip's Translation of the London Pharmacopœia.* On a former occasion we expressed a high opinion of Mr. Phillips's translation of the Pharmacopœia, or what with more justice might be called a commentary upon the tests and processes contained in it, accompanied with a translation. The London College are under deep obligations to this author for his clear and valuable illustrations: indeed many tests and processes would be unintelligible to students without them. This is well exemplified, conversely in the case of the Edinburgh Pharmacopœia, which in many particulars must be imperfectly understood, and on this account will be less valuable to the student until this deficiency is supplied. Mr. Phillips has also in many cases given the additional characters of the agents treated of, which are uniformly the result of the most modern chemical researches; and in respect of pharmaceutical accuracy and illustrations his translation of the pharmacopœia is unrivalled by any similar British work.

*Dunglison's New Remedies.* Dr. Dunglison's work, like that of Mr. Phillips, has gone through several editions within a very short period, which shows the high estimation in which it is held by the profession. His pharmaceutical, toxicological and therapeutic researches are very elaborate and extensive, and prove that he is familiar with all the modern discoveries; whilst his own practical deductions are in general cautious and very judicious. The work contains a complete account of almost every substance that has been recently introduced into practice, some of the articles being very fully described, such as iodine, creosote, &c.; it is well adapted for the guidance of those who wish to prescribe agents that they are imperfectly acquainted with. His formulæ are numerous and various, being chiefly derived from those authors who have written respecting the agents described. Some very useful remarks are also made upon subjects not generally included in pharmacological works; such as acupuncture, electro-puncture, compression, counter-irritation, moxa, galvanism, catheterism of the eustachian tube, &c. The present edition, besides some other improvements, contains an account of several entirely new agents, as iodide of ammonia, iodide of arsenic and mercury, cimicifuga, lactate of iron, protocarbonate of iron, monesia, &c. The author has also given a short account in his preface of some other new remedies, although he thinks the testimony in their favour is not sufficient to justify their admission into the body of the work. These are, anthrakokali, a compound of coal and potass, containing in another formula also sulphur, and employed in chronic cutaneous diseases; sulphate of cadmium employed to remove spots from the cornea and in chronic conjunctivitis; gentiana chirayta and paulinia. We regret that our limits forbid our making any extracts from this work, but we cordially recommend it to the notice of our readers.

